# Correction: Promotion of neuroinflammation in select hippocampal regions in a mouse model of perimenopausal Alzheimer’s disease

**DOI:** 10.3389/fmolb.2025.1684511

**Published:** 2025-09-01

**Authors:** Roberta Marongiu, Jimcy Platholi, Laibaik Park, Fangmin Yu, Garrett Sommer, Clara Woods, Teresa A. Milner, Michael J. Glass

**Affiliations:** ^1^ Feil Family Brain and Mind Research Institute, Weill Cornell Medicine, New York, NY, United States; ^2^ Neurological Surgery Department, Weill Cornell Medicine, New York, NY, United States; ^3^ Genetic Medicine Department, Weill Cornell Medicine, New York, NY, United States; ^4^ Anesthesiology Department, Weill Cornell Medicine, New York, NY, United States

**Keywords:** amyloid beta, astrocyte, microglia, ovarian failure, pyramidal cell

Author Laibaik Park was erroneously spelled as Laibak Park.

The original article has been updated.

