# Supplementary figures and images for: Promotion of neuroinflammation in select hippocampal regions in a mouse model of perimenopausal Alzheimer’s disease
Source: Front Mol Biosci. 2025 May 14;12:1597130. doi: 10.3389/fmolb.2025.1597130 (PMC12116374; doi:10.3389/fmolb.2025.1597130)

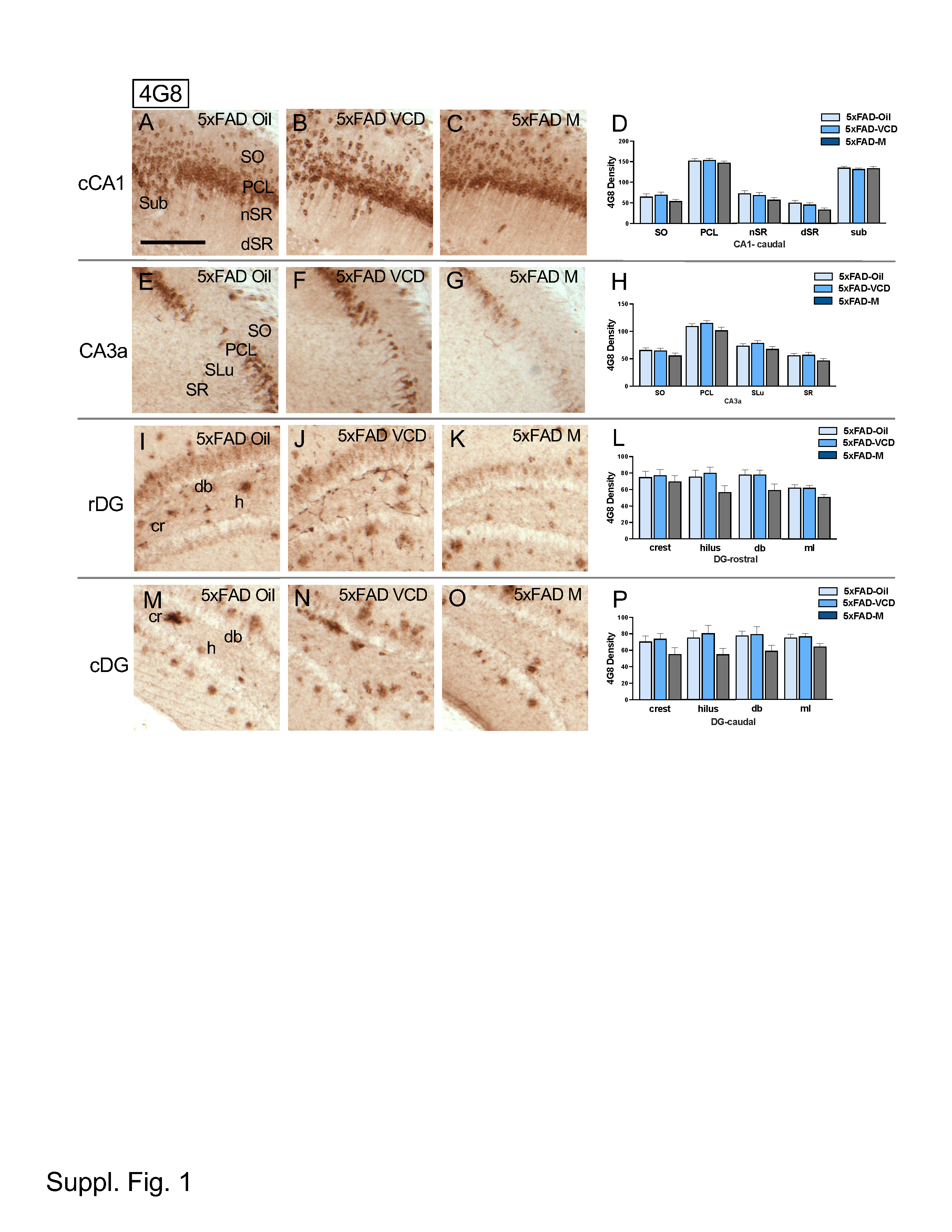

Supplement: Supplementary file 1 [file Image1.tiff]

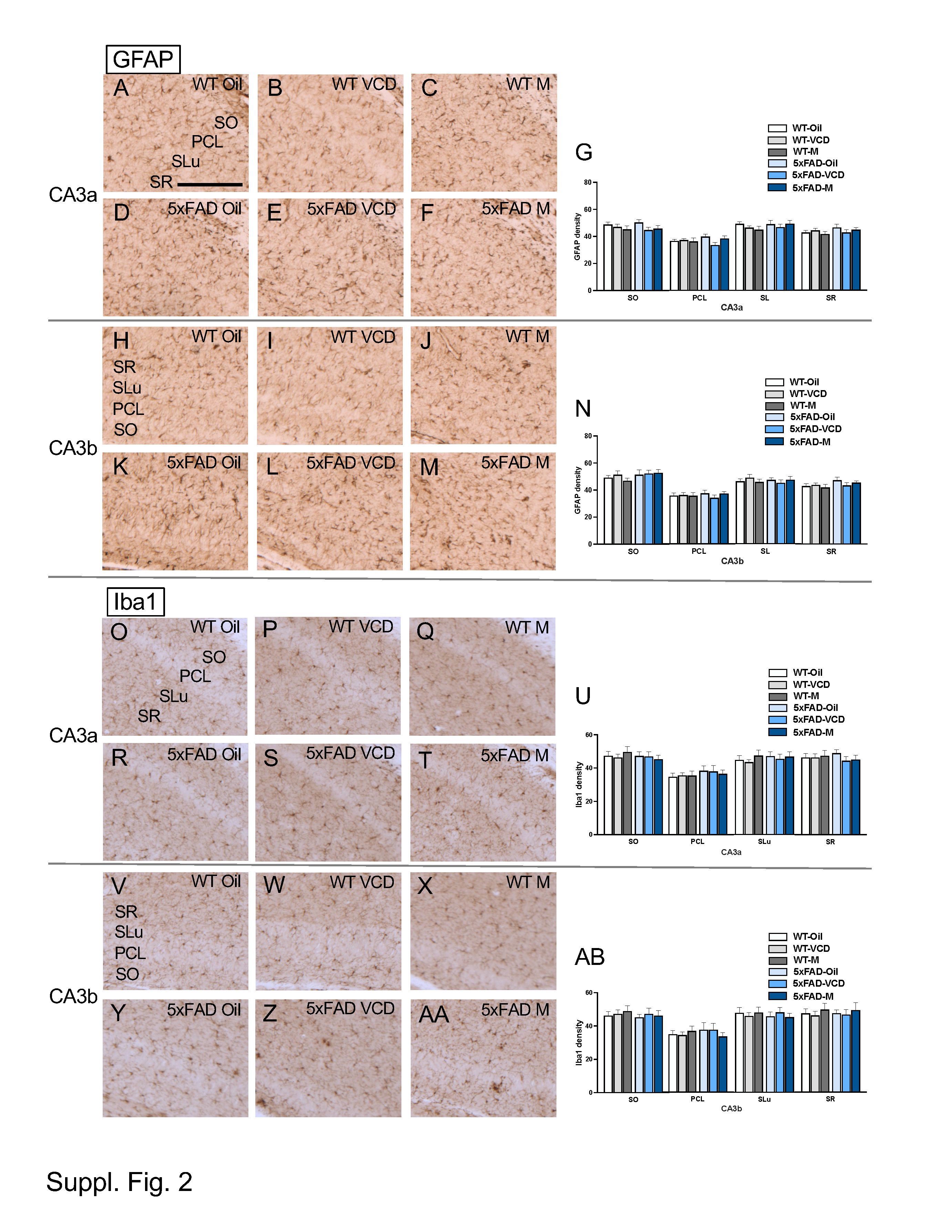

Supplement: Supplementary file 2 [file Image2.tiff]
